# A hierarchical vascularized engineered bone inspired by intramembranous ossification for mandibular regeneration

**DOI:** 10.1038/s41368-022-00179-z

**Published:** 2022-06-22

**Authors:** Xin Ye, Jianxiang He, Shaolong Wang, Qianglong Han, Dongqi You, Bin Feng, Feiya Zhao, Jun Yin, Mengfei Yu, Huiming Wang, Huayong Yang

**Affiliations:** 1 Key Laboratory of Oral Biomedical Research of Zhejiang Province, Stomatology Hospital, School of Stomatology, Zhejiang University School of Medicine, Zhejiang Provincial Clinical Research Center for Oral Diseases, Hangzhou, China; 2 The State Key Laboratory of Fluid Power and Mechatronic Systems, Key Laboratory of 3D Printing Process and Equipment of Zhejiang Province, School of Mechanical Engineering, Zhejiang University, Hangzhou, China

**Keywords:** Biomedical materials, Bioinspired materials

## Abstract

Mandibular defects caused by injuries, tumors, and infections are common and can severely affect mandibular function and the patient’s appearance. However, mandible reconstruction with a mandibular bionic structure remains challenging. Inspired by the process of intramembranous ossification in mandibular development, a hierarchical vascularized engineered bone consisting of angiogenesis and osteogenesis modules has been produced. Moreover, the hierarchical vascular network and bone structure generated by these hierarchical vascularized engineered bone modules match the particular anatomical structure of the mandible. The ultra-tough polyion complex has been used as the basic scaffold for hierarchical vascularized engineered bone for ensuring better reconstruction of mandible function. According to the results of in vivo experiments, the bone regenerated using hierarchical vascularized engineered bone is similar to the natural mandibular bone in terms of morphology and genomics. The sonic hedgehog signaling pathway is specifically activated in hierarchical vascularized engineered bone, indicating that the new bone in hierarchical vascularized engineered bone underwent a process of intramembranous ossification identical to that of mandible development. Thus, hierarchical vascularized engineered bone has a high potential for clinical application in mandibular defect reconstruction. Moreover, the concept based on developmental processes and bionic structures provides an effective strategy for tissue regeneration.

## Introduction

The mandible is one of the most prominent bones of the craniomaxillofacial region and is closely related to the preservation of facial appearance and functions, such as chewing, pronunciation, and speech.^1^ Due to the large volume and irregular shape of massive mandibular defects, they are difficult to reconstruct appropriately. Furthermore, stress concentration resulting from mastication leads to bone resorption and fractures. Therefore, massive mandibular defect reconstruction is highly challenging.^2,3^ Fortunately, advancements in, for instance, lab-constructed tissue-engineered bone provide promising options for individuals with these defects.^4^ Recently, related research has mainly focused on three aspects: scaffold materials, seed cells, and growth factors, while very few studies have focused on reconstructing the physiological mandibular structure from the perspective of developmental processes.^5^

During development, the neural crest-derived mandible mainly undergoes intramembranous ossification,^6^ which starts with the avascular soft condensation of mesenchymal cells, followed by angiogenesis.^7^ The capillary-like structures invade the avascular condensation of mesenchymal cells, and then, the vascularized bone is formed, which eventually grows into the mandible.^8^ The bone tissue formed by intramembranous ossification increases the mandibular mechanical strength at an early stage and shortens the entire repair period when compared with those of bone tissue formed by endochondral ossification.^9,10^ Regarding the mandibular anatomy, the inferior alveolar artery supplies branches to the mandibular body in an upward manner.^11^ Thus, angiogenesis plays an important role in mandibular development and defect reconstruction.

GelMA and Matrigel are widely used for cell encapsulation and vascular network formation, which regulate capillary formation and invasion and provide nutrients and oxygen to surrounding cells during mandibular regeneration.^12–15^ However, the vehicle hydrogel cannot withstand the strain and stress generated by mastication. To obtain better mechanical properties, hydrogels have been reinforced with nanofibers, microfibers, and scaffolds.^16–18^ Among these methods, scaffolds fabricated by 3D printing can match the irregular shape in mandibular reconstruction. Conventional 3D printing biomaterials, such as polycaprolactone (PCL) and poly(lactic acid) (PLA) are prone to stress concentration, and bone absorption or even fracture could occur under occlusal loading, which does not satisfy the requirements for mandible reconstruction.^19–21^ Polyion complexes (PIC), synthesized from oppositely charged polyelectrolytes, have recently been reported to have high toughness and good biocompatibility.^22–24^ Thus, the combined application of PIC scaffolds and vehicle hydrogels is an ideal choice for mandibular reconstruction.

**Scheme 1:**
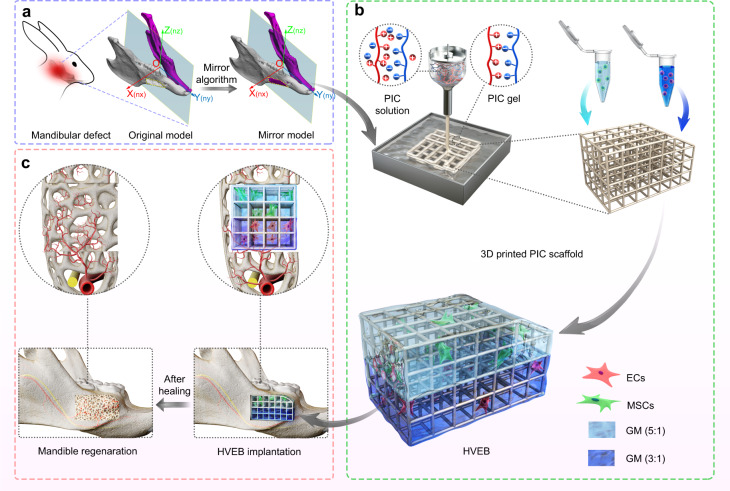
Schematic depicting the hierarchical vascularized engineered bone (HVEB) for mandibular reconstruction. **a** Computed tomography (CT) images obtained from the defect area are first used to reconstruct the 3D model of the mandible; then, the defect area is separated from the bone, and the prosthesis is designed via a mirror algorithm. **b** The design of the external shape and structure of the scaffold is according to the prosthesis. The fundamental scaffold is constructed precisely using 3D printing technology with PIC materials. Different concentration gradient vehicle hydrogels loaded with MSCs and ECs, respectively, are perfused into PIC fundamental scaffold successively. This forms an angiogenesis module and an osteogenesis module hierarchically, which is named HVEB. **c** The HVEB is transplanted into animal mandibular critical defect models for bone regeneration

Herein, we propose a strategy for the assembly of a hierarchical vascularized engineered bone (HVEB) inspired by intramembranous ossification to repair critical mandibular defects (Scheme 1). Endothelial cells (ECs) formed a capillary network in the angiogenesis module and successfully invaded the osteogenesis module, simulating the spatiotemporal structure of intramembranous ossification. The sonic hedgehog (Shh) signaling pathway, which is essential for intramembranous ossification, was activated in HVEB. The bone regenerated using HVEB achieved satisfactory healing and formed a similar morphology and genomics to those of the natural mandible. More importantly, this system regards mandibular defect regeneration as a pathological developmental process and reconstructs the mandible from a natural developmental perspective.

## Results and discussion

### PGM (5:1) achieves excellent stress dispersion ability and biocompatibility

**Fig. 1:**
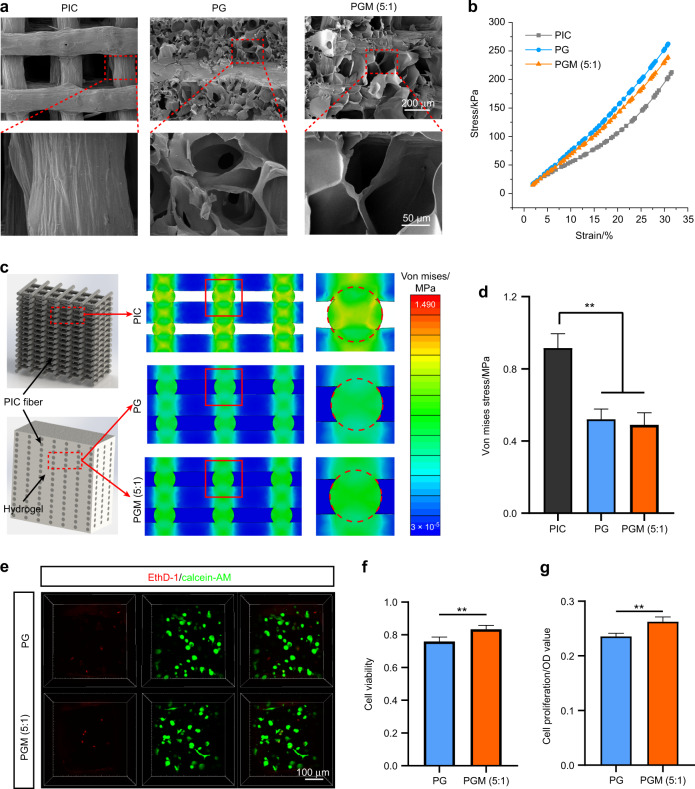
PGM (5:1) exhibits excellent stress dispersion ability and biocompatibility. **a** SEM images for PIC, PG, and PGM (5:1). **b** Compressive stress–strain curves for PIC, PG, and PGM (5:1). **c** Stress distribution for the PIC, PG, and PGM (5:1) simulated using FEM under boundary conditions similar to experimental tests. **d** Von mises stress for PIC, PG, and PGM (5:1) (*n* = 3, **P < 0.01). **e** Live/Dead staining of cells cultured in PG and PGM (5:1) after day 1. **f** Quantitative results of cellular viability of cells cultured in PG and PGM (5:1) after day 1 (*n* = 5, **P < 0.01). **g** Quantitative results of cellular proliferation of cells cultured in PG and PGM (5:1) after day 1 (*n* = 5, **P < 0.01)

To construct a regenerative system that was adapted to mandibular occlusion function and guaranteed cell survival, we combined the PIC scaffold with GelMA or modified GelMA [ratio of GelMA: Matrigel is 5:1, GM (5:1)], which were named as PG and PGM (5:1), respectively. The porous vehicle hydrogel was evenly distributed among the fibers of the PIC scaffold (Fig. 1a). The PIC scaffold of PG and PGM (5:1) showed better tensile strength than that of single fiber and cross-fiber (Supplementary Fig. 1). Based on the results of compressive tests, the compressive moduli of PIC, PGM (5:1), and PG scaffolds were 0.60, 0.70, and 0.72 MPa, respectively (Fig. 1b), which indicated that the compressive modulus of PIC scaffolds was improved by adding GelMA and Matrigel into the scaffolds. Based on the numerical results of the finite element method (FEM), we found that the maximum value of Von Mises stress in the PIC scaffold was much higher than that in the PG and PGM (5:1) scaffolds (Fig. 1c), which indicated that the PG and PGM (5:1) scaffolds had a stronger ability to disperse stress than the PIC scaffold. Quantitative analysis of FEM results revealed that the maximum stress always exited in the nodes of the scaffolds, and the stress in the nodes of PG and PGM (5:1) scaffolds was much less than the stress in the nodes of the PIC scaffold (Fig. 1d).

To assess the biocompatibility of PG and PGM (5:1), mesenchymal cells (MSCs) were encapsulated in PG and PGM (5:1). MSCs cultured in PGM (5:1) had higher cell viability than those cultured in PG (Fig. 1e, f). The cell proliferation results also confirmed that PGM (5:1) was more conducive to MSC proliferation than PG (Fig. 1g). Therefore, PGM (5:1) was selected for subsequent experiments, since it provided a microenvironment with suitable mechanical properties and biocompatibility for cell survival.

### PGM (5:1) promotes the spread of seed cells and the formation of the capillary-like network of ECs

**Fig. 2:**
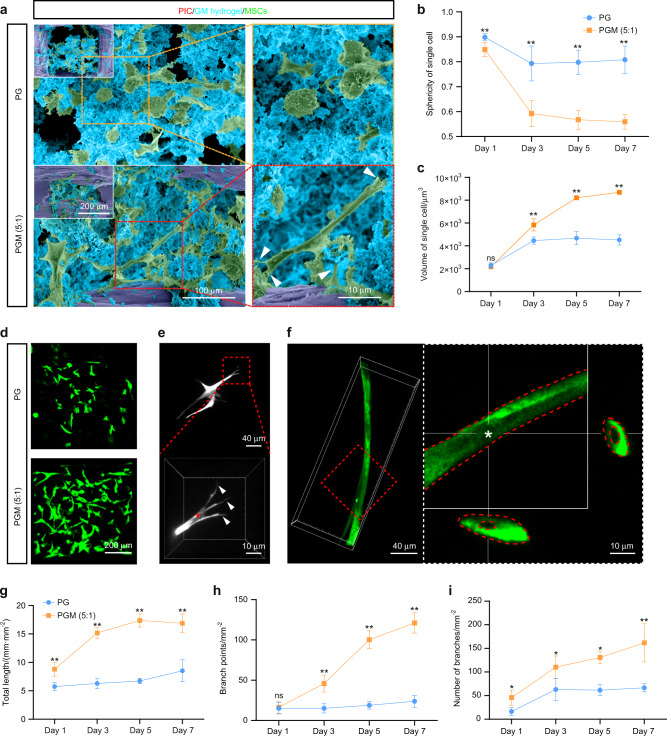
PGM (5:1) promotes the spread of seed cells and formation of the capillary-like network of ECs. **a** Representative SEM images of MSCs cultured in PG and PGM (5:1) at day 7. Cells were stained green, GM hydrogel was stained blue, and the PIC scaffold was stained purple. **b**, **c** Quantitative analysis of the extent of spread of MSCs in PGM (5:1) and PG including sphericity (**b**) and volume (**c**) of a single cell (*n* > = 50, *P < 0.05, **P < 0.01). **d** Phenotype of encapsulated ECs in PG and PGM (5:1) after 7 days. **e** Maximum intensity projections of confocal images show the sprouts (indicated with arrows) and lumens (indicated with asterisks) of ECs encapsulated within PG and PGM (5:1). **f** Representative confocal maximum intensity projection with orthogonal views (top and right side) of luminal structures (indicated with asterisks) in PGM (5:1). **g**–**i** Quantitative analysis of the extent of capillary-like network formation: total capillary-like length per unit of area (**g**), the branch points per unit of area (**h**), and the number of branches per unit of area (**i**) (*n* = 4, *P < 0.05, **P < 0.01)

To fabricate mesenchymal cell condensation, MSCs were encapsulated in PG and PGM (5:1). We observed the morphology of MSCs cultured in PG and PGM (5:1) on day 7 to investigate their growth status. Scanning electron microscopy (SEM) revealed that MSCs were wrapped with GelMA or GM (5:1) and filled in the gaps of the PIC fibers (Fig. 2a). MSCs spread more freely and formed more filopodia in PGM (5:1) than in PG. Laser scanning confocal microscopy (LSCM) images revealed that MSCs encapsulated in PGM (5:1) showed subtle protrusions as early as the first day of culture, while they remained round in PG (Supplementary Fig. 2). The results of single-cell sphericity (Fig. 2b) and volume calculations (Fig. 2c) also demonstrated that MSCs spread better in PGM (5:1) than in PG.

The main difference in the MSC culture between PGM (5:1) and PG was in the matrix density, which influenced the nutrient, oxygen, and cytokine exchange.^25,26^ GM (5:1) is the main component of PGM (5:1), which has an appropriate matrix density, leading to optimized stiffness, pore size, and porosity (Supplementary Figs. 3a–d and 4). Diffusional permeability testing revealed that GM (5:1) had a better diffusion coefficient than GelMA (Supplementary Fig. 3e), which was more beneficial for cellular growth and spread. The degradation property of GM (5:1) was similar to that of GelMA (Supplementary Fig. 3f, g).

Vascularization is critical for successful mandible reconstruction.^27,28^ To generate the vascular network, ECs were encapsulated in PGM (5:1) or PG. ECs in PGM (5:1) also showed a better spread morphology than those in PG (Fig. 2d). The vascular bed was observed in PGM (5:1) at day 7, and the extent of network formation in PGM (5:1) was improved, with significantly increased total network length (Fig. 2g), branch points (Fig. 2h), number of branches (Fig. 2i), and average branch length (Supplementary Fig. 5). Immunofluorescence staining revealed a higher expression of the angiogenesis marker CD31 (PECAM-1) in ECs encapsulated in PGM (5:1) (Supplementary Fig. 6). Vascular morphogenesis is initiated by the ECs interacting with the matrix and forming void spaces called vacuoles, which finally develop into a lumen. With ECM remodeling, the sprouting and branching of ECs results in the formation of a nascent vasculature.^29,30^ The sprouting, branching (Fig. 2e), and formation of vacuoles (Fig. 2f) in PGM (5:1) confirmed vascular morphogenesis. These results indicated that PGM (5:1) could provide an appropriate environment for seed cells and form capillary-like networks.

### PGM concentration gradient regulates the directional migration of ECs

**Fig. 3:**
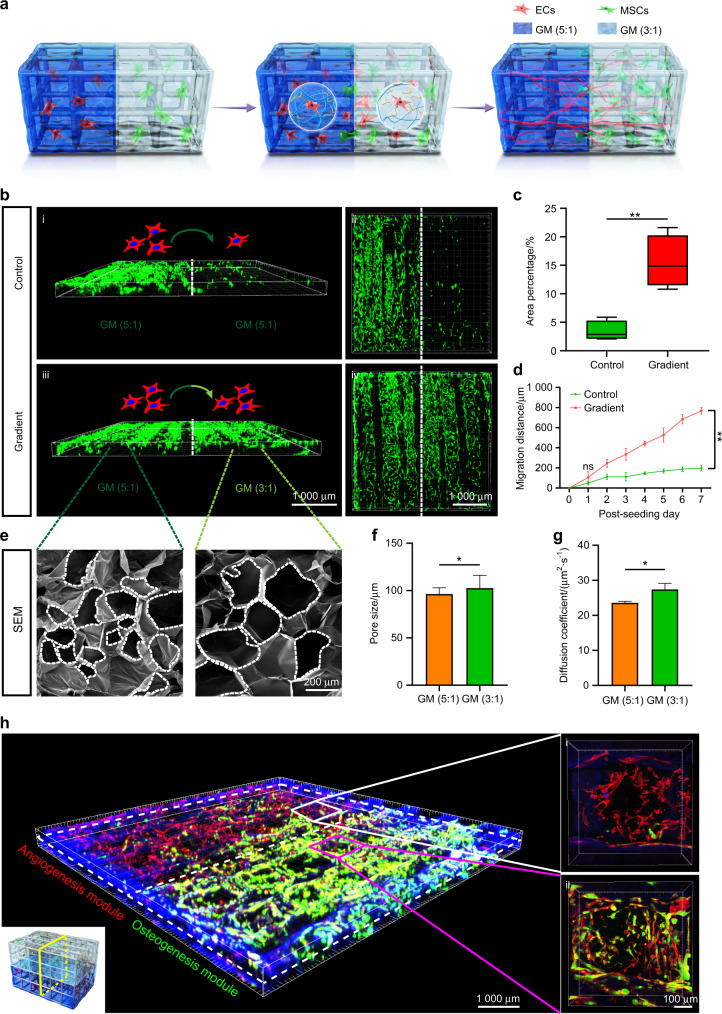
The PGM concentration gradient regulates the directional migration of ECs. **a** Schematic description of migration of ECs from GM (5:1) to GM (3:1) and vascular network formation. **b** 3D LSCM images of distribution of ECs after 7 days of migration from GM (5:1) to GM (3:1) (gradient group) or GM (5:1) (control group). **c** Area percentage of ECs in GM (3:1) (gradient group) or GM (5:1) (control group) migrated from GM (5:1) on day 7 (*n* = 5, **P < 0.01). **d** Migration distance of ECs in GM (3:1) (Gradient group) or GM (5:1) (control group) migrated from GM (5:1) at days 1, 2, 3, 4, 5, 6, and 7 (*n* = 5, **P < 0.01). **e** Morphology of GM (5:1) and GM (3:1), obtained using SEM. White dotted line defines pore boundary. **f**, **g** Pore size (**f**) and diffusion coefficient (**g**) of GM (5:1) and GM (3:1) (*n* = 3, *P < 0.05, **P < 0.01). **h** 3D LSCM images of HVEB, including angiogenesis module and osteogenesis module

In intramembranous ossification, as the diffusion of oxygen is limited to a length scale of ~100–200 μm from blood vessels, the process of capillary invasion of avascular mesenchymal cell condensation is crucial.^31,32^ To mimic capillary invasion, we constructed gradient modules of PGM (5:1) and PGM (3:1) for EC migration (Fig. 3a). In brief, GM (5:1) encapsulated with ECs and GM (3:1) encapsulated with MSCs were serially perfused into the PIC scaffold to form a hierarchical capillary-like network and mesenchymal cell condensation.

ECs successfully migrated from GM (5:1) to GM (3:1) (Fig. 3b). The increased area of ECs in the gradient group was significantly higher than that in the control group, showing a better migration of ECs in the gradient group (Fig. 3c). The migration distance of ECs was significantly different between the gradient and control groups from day 2 (Fig. 3d). Cell migration is regulated by biophysical and chemical properties, including matrix density and growth factors.^33–37^ First, GM (3:1) had a larger pore size (Fig. 3e, f) and porosity (Supplementary Fig. 7) than that of GM (5:1). The increase in hydrogel pore size could improve cell migration and vascularization.^38,39^ Second, the diffusion coefficient of GM (3:1) was significantly higher than that of GM (5:1) (Fig. 3g and Supplementary Fig. 8), indicating an easier transport of substances, including growth factors, nutrients, and oxygen in GM (3:1).^26,36^ Third, GM (5:1) and GM (3:1) differed in the Matrigel content, and the differences in the concentration of cytokines from Matrigel can drive chemotactic cell migration.^34,40^ These different biophysical and biochemical cues between GM (3:1) and GM (5:1) collectively directed the migration and vascularization in the gradient vehicle hydrogel, fabricating an HVEB in vitro (Fig. 3h). The vascular bed was observed in the angiogenesis module (Fig. 3hi), and the capillary network was formed in the osteogenesis module (Fig. 3hii), realizing the vascularization of the whole engineered bone. Ultimately, HVEB successfully simulated the process of intramembranous ossification, showing great potential for in vivo mandibular regeneration.

### Mandible reconstructed using HVEB forms a hierarchical vascular network including inferior alveolar artery, arterioles, and capillaries

**Fig. 4:**
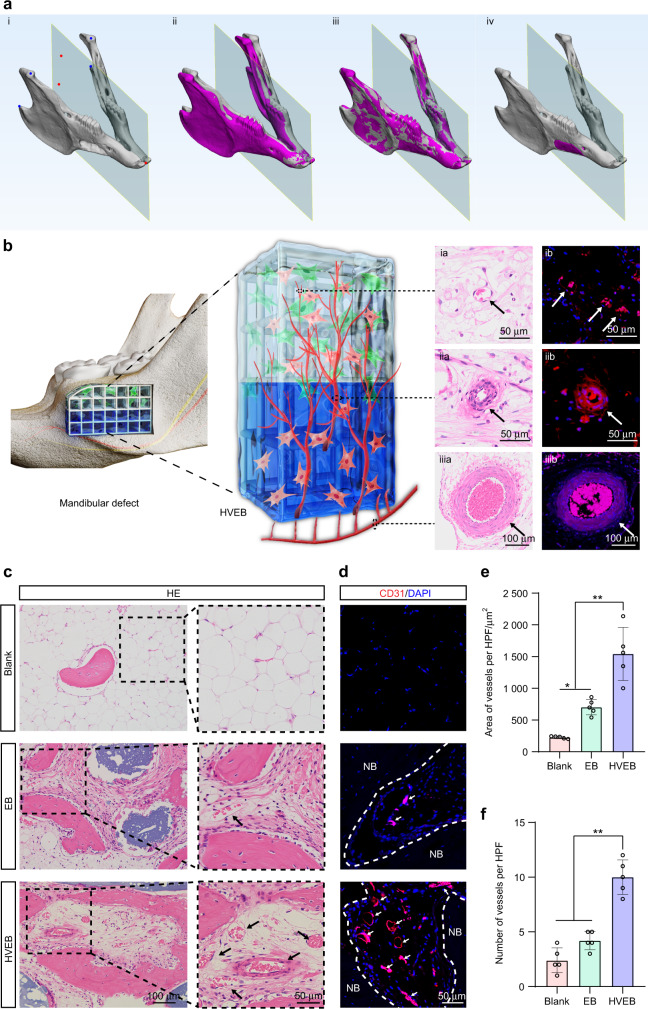
The mandible reconstructed using HVEB forms a hierarchical vascular network, including the inferior alveolar artery, arterioles, and capillaries.**a** Three-dimensional model reconstruction of the mandibular defect. **b** Schematic depiction of three-dimensional vascular supply network formed in HVEB after implantation in the mandibular defect. **c** Hematoxylin and eosin (HE) staining sections of neovascularization in the mandibular defect area. Black arrows point toward newly formed vessels. **d** Immunofluorescence images for CD31 of newly formed vessels. White arrows point toward newly formed vessels. White dotted lines define the boundary of the newly formed bone. NB new bone. **e**, **f** Quantification of area (**e**) and number (**f**) of newly formed vessels per HFP (×200). (*n* = 5, *P < 0.05, **P < 0.01)

We constructed a critical mandibular defect model in vivo to demonstrate the regeneration ability of HVEB. To obtain the 3D model of the defect area, an initial symmetry plane was delineated by the incisor tip, the midpoint of bilateral condyles, and the midpoint of bilateral gonions (Fig. 4aia). The mandible was mirrored with the initial symmetry plane (Fig. 4aii), and the mirrored mandible was globally registered to the original mandible using Procrustes analysis (Fig. 4aiii). Finally, the registered mandible was subtracted from the original mandible with the Boolean algorithm to obtain the model best fitting the defect (Fig. 4aiv). The mandibular defect was repaired using HVEB, engineered bone without hierarchical vascularization (EB group), engineered bone without seed cells (no cell group), or without using implants (blank group), respectively.

A hierarchical vascular network consisting of an inferior alveolar artery (Fig. 4biiia), arterioles (Fig. 4biia), and capillaries (Fig. 4bia) was only observed in the HVEB group. It was similar to the blood supply network in the natural mandible, which was supplied by an upward vascular network composed of the inferior alveolar artery and its branches.^11^ Histological analysis was carried out to assess neovascularization in the defects of different groups after 12 weeks. More neovascular tissue was observed in the HVEB group compared to that in the blank, no cell, and EB groups (Fig. 4c and Supplementary Fig. 9a). Immunofluorescence staining showed that the HVEB group had significantly higher CD31-positive vessels than other groups (Fig. 4d and Supplementary Fig. 9b). The quantitative results of newly formed vessels confirmed that the HVEB group had the most successful vascularization among all groups (Fig. 4e, f and Supplementary Fig. 9c, d). These nascent vessels were located between the newly formed trabecular bones and were functional with erythrocytes appearing in the lumen structures. The neovessel guaranteed adequate oxygenation, nutrient delivery, and removal of waste products, which accelerated the formation of new bone and blood vessel tissues. These results indicated that HVEB formed a hierarchical vascular network.

### Mandible reconstructed using HVEB achieves better bone regeneration

**Fig. 5:**
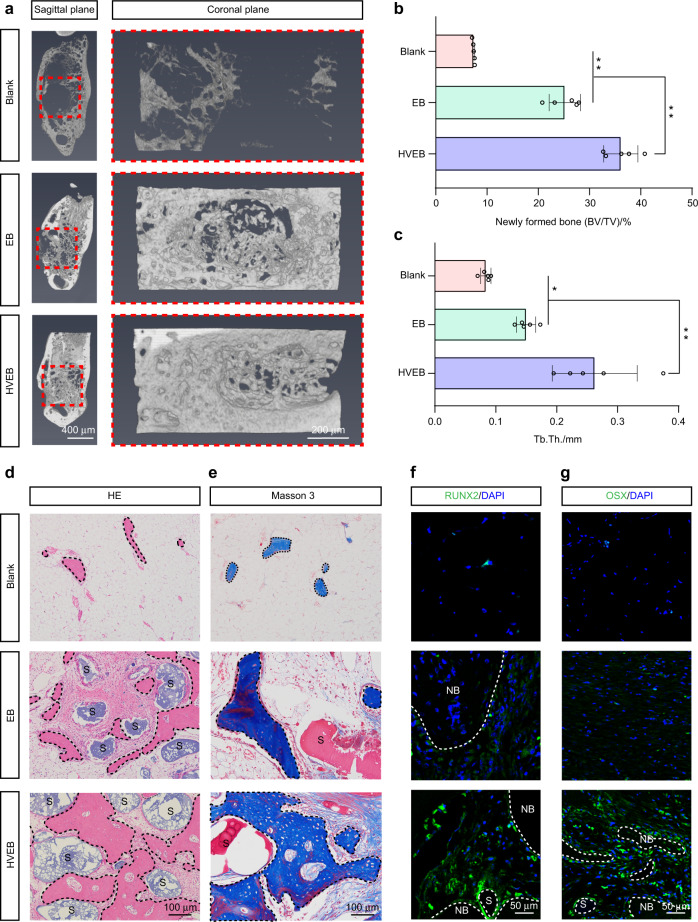
The mandible reconstructed using HVEB achieves better bone regeneration. **a** Representative 3D images of coronal sections of a mandibular defect in a rabbit model after implantation of tissue-engineered bone materials. Red boxes define the defect margins. **b**, **c** Quantified results of BV/TV (**b**) and Tb.Th. (**c**) from micro-CT. (*n* = 5, *P < 0.05, **P < 0.01). **d**, **e** HE (**d**) and Masson’s trichrome (**e**) staining results of bone regeneration in defect area. Black dotted lines define the boundary of the newly formed bone. S: PIC scaffold. **f**, **g** Representative immunofluorescence images of newly formed bone tissue stained with osteogenesis markers RUNX2 (**f**) and OSX (**g**). White dotted lines define the boundary of the newly formed bone

Micro-computed tomography (micro-CT) results revealed that HVEB implantation achieved the best bone defect healing, and the newly formed bone tissue experienced active remodeling after 12 weeks, followed by the EB and no cell groups (Fig. 5a and Supplementary Fig. 10a). The blank group showed little bone tissue regeneration. The quantified results of newly formed bone volume/total volume (BV/TV) (Fig. 5b and Supplementary Fig. 10f), trabecular thickness (Tb.Th.) (Fig. 5c and Supplementary Fig. 10g), and trabecular spacing (Tb.Sp.) (Supplementary Figs. 10h and 11) confirmed the observations shown in Fig. 5a. Hematoxylin and eosin (HE) and Masson’s trichrome staining revealed more new bone formation in the HVEB group compared to that in the EB, no cell, and blank groups (Fig. 5d, e and Supplementary Fig. 10b, c). The quantitative results of newly formed bone demonstrated successful defect healing in the HVEB group (Supplementary Fig. 12). Moreover, higher expression of the osteogenic marker genes *RUNX2* and *OSX* was observed in the HVEB group (Fig. 5f, g and Supplementary Fig. 10d, e). The tissue regenerated in the HVEB group consisted of trabecular bone instead of cartilage tissue, which is usually observed in the process of endochondral ossification.^41^ Skipping the cartilage formation stage and directly forming trabecular bone can greatly reduce the bone healing period and strengthen the mechanical properties at the early stages. Combining these histological results with the highly ordered vascular tissue structure in HVEB, we verified that HVEB achieved good mandible repair effects and speculated that its bone regeneration process may be intramembranous ossification.

### Gene expression profile of the regenerated bone tissue repaired by HVEB is similar to that of the natural mandible and possibly undergoes intramembranous ossification via the Shh pathway

**Fig. 6:**
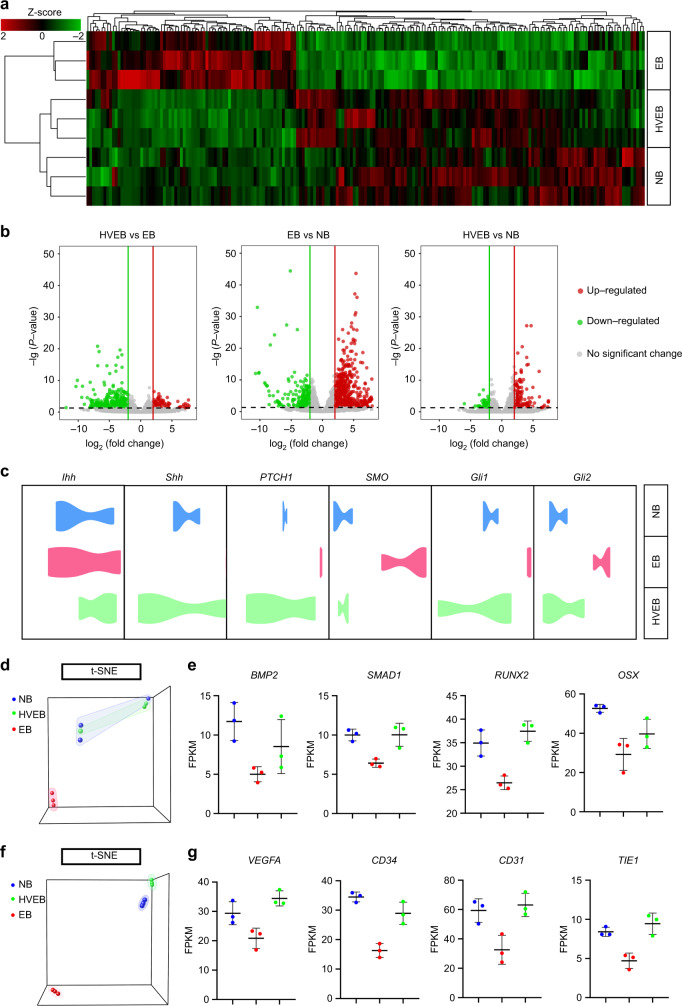
Gene expression profile of the regenerated bone tissue repaired by HVEB is similar to that of the natural mandible and possibly undergoes intramembranous ossification via the Shh pathway. **a** RNA-seq analysis of normal mandibular bone (NB), newly formed tissue regenerated by EB and HVEB. Gene expression profiles are presented using a heatmap. **b** Volcano plots of differentially expressed genes in NB, EB, and HVEB groups. **c** Relative expression levels of genes of Hedgehog signaling pathway (*IHH, SHH, PTCH1, SMO, GLI1*, and *GLI2*) in the NB, EB, and HVEB groups. **d** t-SNE visualization of osteogenesis genes for NB, EB, and HVEB groups. **e** Osteogenesis gene signatures of *BMP2, SMAD1, RUNX2*, and *OSX* based on the relative expression levels in NB, EB, and HVEB groups. **f** t-SNE visualization of angiogenesis genes in NB, EB, and HVEB groups. **g** Angiogenesis gene signatures of *VEGFA, CD34, CD31*, and *TIE 1* based on the relative expression levels in NB, EB, and HVEB groups (*n* = 3)

To further evaluate how functionally similar the mandibles regenerated by HVEB or EB were to the natural mandible and the method of bone regeneration, we performed RNA sequencing (RNA-seq) to compare the gene expression profiles of bone tissue from the mandible of non-surgical rabbits, HVEB, and EB. The cells extracted from the HVEB group had more similar expression of genes involved in osteogenesis and vascularization to that of the natural mandibular bone (NB) group. In contrast, more genes involved in adipogenesis and fibrogenesis were expressed in the EB group (Fig. 6a).

The endogenous cells in the EB group had approximately 1200 upregulated genes and 800 downregulated genes relative to those of the NB group, whereas there were only 200–300 differentially expressed genes between the cells in the NB and HVEB groups (Fig. 6b). Moreover, t-distributed stochastic neighbor embedding (t-SNE) analysis showed similar results (Fig. 6d). The Hedgehog (Hh) signaling pathway plays an important role in bone development and regeneration.^42^ Shh, a homolog of Hedgehog closely related to intramembranous ossification and endochondral ossification, was highly expressed in the HVEB group compared to its levels in the EB group (Fig. 6c).^43^ At the same time, the expression of the downstream genes Patched1 (*Ptch1*), Smoothened (*SMO*), and *Gli* of the Hh signaling pathway were all upregulated in the HVEB group (Fig. 6c), indicating that this pathway was activated to regulate bone tissue regeneration. However, Indian Hedgehog (*Ihh*), another Hedgehog homologous gene, which has been proven essential in endochondral ossification but is not required in intramembranous ossification, was found at low expression levels in the HVEB group (Fig. 6c).^44–46^ This evidence suggests that the regenerated bone obtained using HVEB may undergo intramembranous ossification rather than endochondral ossification during regeneration. Osteogenesis markers, such as *BMP2, SMAD1, RUNX2*, and *OSX* were expressed at higher levels in the endogenous cells of the HVEB group than in those of the EB group (Fig. 6e).^47^ Shh also plays a key role in vascular development and has been used to fabricate engineered blood vessels to enhance bone tissue formation.^48^ Consistent with the t-SNE analysis of osteogenesis genes, t-SNE analysis of angiogenesis genes showed that vascular regeneration-related gene expression in the HVEB group was similar to that in the natural bone tissue (Fig. 6f). Angiogenesis marker genes, such as *VEGFA, CD34, CD31*, and *TIE 1*, showed increased expression in the endogenous cells of the HVEB group compared to that in the endogenous cells of the EB group (Fig. 6g).^49^ Meanwhile, adipogenesis and fibrogenesis-related gene expression in the HVEB group was similar to that in the natural bone tissue but lower than that in the EB group (Supplementary Fig. 13a, b). These results demonstrated that HVEB had a similar gene expression profile to that of the natural mandible tissue. Furthermore, the gene expression profile of HVEB indicated that the new bone tissue may undergo intramembranous ossification through the Shh signaling pathway.

## Conclusions

In summary, we fabricated an HVEB based on a PIC scaffold, gradient vehicle hydrogel, and seed cells, inspired by the process of intramembranous ossification. The hierarchical osteogenesis module and angiogenesis module spatially constructed the mesenchymal cell condensation and capillary structure, respectively. We successfully realized the process of capillary invasion into the mesenchymal cell condensation, which usually occurs in intramembranous ossification. In the rabbit mandibular critical defect model, HVEB, which likely underwent intramembranous ossification through the Shh signaling pathway, displayed a high efficiency in vascularization and bone regeneration. We anticipate that the reported method will be applicable for clinical translation in the treatment of massive mandibular defects and provide a new perspective of tissue engineering design inspired by the natural developmental process.

## Materials and methods

### Materials

The procedure of preparation of PIC has been described in our previous work. In brief, in the presence of 0.05 mol % (relative to the monomer) 2-oxoglutaric acid under UV light irradiation (365 nm wavelength, 7.5 mW·cm^−2^) for 8 h, the precursor aqueous solutions of 1 mol·L^−1^ Sodium pstyrenesulfonate (NaSS) and 1 M 3-(methacryloylamino)propyl-trimethylammonium chloride (MPTC) were polymerizied and synthesized as PNaSS and PMPTC. PNaSS and PMPTC, respectively. Solutions with equal volume were slowly dripped into deionized water and stirred for 30 min, forming PIC precipitates, which were dissolved in sodium chloride solution to form the PIC solution.^24^ The GelMA were prepared as previously described.^24,50^ Before use, freeze-dried GelMA (5% w/v final) was dissolved with the photoinitiator (0.5 w/v%, lap; StemEasy, China) at 37 °C. GM was made by adding Matrigel (Corning, USA) to GelMA at the different volume ratios of 1:3 [GM (3:1)] or 1:5 [GM (5:1)].

### Preparation and characterization of PIC, PG, and PGM (5:1)

A custom 3D printing system, composed of an extrusion-based dispersion system and a 3D moving stage, was used to print PIC scaffolds. The extrusion system consisted of an air pump, a pressure controller, and a heating syringe with temperature control. In the printing process, the PIC precursor solution was loaded into a selected syringe and pneumatically extruded into a deionized water container at 25 °C, where the PIC solution rapidly gelated into a solid gel.

The PG/PGM was constructed by injecting the GelMA/GelMA-Matrigel precursor into the PIC scaffold and exposed to UV radiation (365 nm, 8 mW·cm^−2^ UV) for 7 s at 25 °C. The microstructures of the PIC, PG, and PGM (5:1) were characterized using SEM (Nova Nano 450, FEI, USA). The surface and cross-sectional porosity and pore size of GM (3:1) and GM (5:1) were determined using ImageJ (NIH, USA). The compressive tests were performed on PIC, PG, and PGM (5:1) having a volume of 5 × 5 × 5 mm^3^ with a compressive velocity of 2.5 mm·min^−1^ using ElectroForce (TA Instruments, USA). The compressive modulus was calculated from the slope of the linear region in the 0%–15% strain range of the stress–strain curves. The tensile tests were performed on the PIC scaffold, single fiber, and cross-fiber using ElectroForce (TA Instruments, USA). Uniaxial stretch was performed at the rate of 1 mm·min^−1^ until the samples broke. The enzymatic degradation properties of GelMA, GM (5:1), GM (3:1), and Matrigel were tested in PBS containing 2 U·mL^−1^ collagenase II (Gibco, USA) at 37 °C. Hydrogels were incubated with collagenase II at 37 °C for 1, 2, 3, and 4 h. At each time point, the collagenase solution was removed. The natural degradation properties of GelMA, GM (5:1), GM (3:1), and Matrigel were measured in PBS at 37 °C for 1, 2, 3, and 4 weeks. The remaining hydrogel was washed with distilled water and all liquid was removed. The hydrogel was lyophilized. The original dry weight is W_o_, and the dry weight after incubation is W_d_. The degree of degradation was determined in percent weight loss using the following equation: Remaining weight (%) = (W_o_ − W_d_)/W_d_ × 100.

### Finite element simulation of PIC, PG, and PGM (5:1) scaffolds

The simulation geometric models of PIC, PG, and PGM (5:1) were obtained using SEM images. For all simulations, PIC, GelMA, and Matrigel were considered as linear elastic isotropic materials with the measured Young’s modulus of 6, 0.031, and 0.028 MPa, respectively, and the Poisson’s ratio for all materials was set as 0.45, owing to their near incompressibility. The numerical model adopted a tetrahedral grid, and the boundary conditions were given according to the uniaxial compression of the scaffold. The displacement of the compression was 20% of the scaffold height. The commercial software ABAQUS (France) was used to complete the compression simulation of PIC, PG, and PGM (5:1).

### In vitro experiments

MSCs and ECs were differentiated from human embryonic stem cells (hESCs, Wicell Research, USA) as reported.^51,52^ The viability of MSCs cultured in PG/PGM was assessed by performing fluorescent live/dead staining and using the CCK-8 assay. For live/dead fluorescent staining, PG/PGM seeded with MSCs after one day of culture were rinsed with phosphate-buffered saline (PBS) and incubated with PBS containing 2 × 10^−3 ^mol·L^−1^ calcein-AM and 4 × 10^−3 ^mol·L^−1^ ethidium homodimer 1 (Invitrogen, USA) at 37 °C for 30 min. The green and red colors represent live and dead cells, respectively, under an inverted laser scanning confocal microscope (Leica, Germany). For the CCK-8 assay, after a 1-, 3-, 5-, and 7-day culture, 200 μL of CCK-8 solution (CCK-8 reagent: medium = 1:10) (Dojindo, Japan) was added into each well. After incubation at 37 °C for 1 h, 100 μL CCK-8 solution was transferred to a new 96-well plate, and the absorbance at 450 nm was measured using a microplate reader (Molecular Devices SpectraMax, USA).

The green fluorescent protein (GFP)-MSCs/GFP-ECs differentiated from GFP-hESCs as reported^53^ were cultured in PG/PGM and observed using an inverted laser scanning confocal microscope (Leica, Germany) at days 1, 3, 5, and 7. Serial optical sections were captured and reconstructed into 3D images, and the cell volume and sphericity were calculated using the Imaris 9.0.1 software (Bitplane, Switzerland).^54^ The morphologies of the MSCs/ECs in PG/PGM were observed using a scanning electron microscope (Nova Nano 450, FEI, USA) as described in our previous study.^55^ ECs were fixed in 4% paraformaldehyde (Biosharp, China) and stained with a mouse anti-CD31 monoclonal antibody (Abcam, UK), rhodamine-phalloidin (Cytoskeleton, USA), and DAPI (Vector, USA).

The total capillary-like length, the average length of capillaries, number of branch points, and number of capillary-like branches were quantified using the ImageJ software (NIH, USA) as reported.^54^

To count the migration of ECs in the PGM gradient, GM (5:1) with GFP-ECs and GM (3:1) with MSCs were added to the PIC scaffold from both sides simultaneously in the gradient group, and GM (5:1) with GFP-ECs and GM (5:1) with MSCs were added to the PIC scaffold from both sides simultaneously in the control group. Fluorescent images were captured using an inverted laser scanning confocal microscope (Leica, Germany).

The HVEB was constructed by adding GM (5:1) with ECs and GM (3:1) with GFP-MSCs into the PIC scaffold from both sides simultaneously. After seven days of culture, HVEB were washed with PBS, fixed with 4% paraformaldehyde (Biosharp, China) for 30 min, and then permeabilized with 0.1% Triton-X 100 (Sigma, USA) in PBS for 5 min. The actin cytoskeleton was stained with rhodamine-phalloidin (Cytoskeleton, USA). Finally, DAPI solution (Vector, USA) was used for nuclear staining for 5 min. Cells were visualized using a confocal laser scanning microscope (Leica, Germany).

### Animal experiments

New Zealand rabbits were anesthetized using intravenous injections of pentobarbital sodium (25 mg·kg^−1^, Sigma, USA) into the lateral ear vein. An injection of 2% lidocaine (CSPC, China) was administered to the operating site. A 2 cm incision was made approximately 1 cm below the lower edge of the mandible body to expose the bone. A segmental critical-size defect (10 × 5 × 5 mm^3^) as carried out in our previous study was prepared in the mandible using low-speed rotary burs under continuous saline irrigation in all animals.^56^ A total of 29 rabbits were used for this experiment. Among them, 20 rabbits were randomly divided into four groups for radiographic and histological tests: HVEB (PGM/ECs/MSCs), EB (PGM/MSCs), no cell (PGM), and blank group. The remaining nine rabbits were randomly divided into three groups for RNA-seq test: HVEB (PGM/ECs/MSCs), EB (PGM/MSCs), and NB (normal bone). The incision was closed in layers using 4–0 resorbable sutures. The animals received prophylactic antibiotics at the time of surgery and for three days postoperatively. The animals were killed by a lethal dose of barbiturate after a healing period of 12 weeks.

For radiographic and histological tests, fresh mandibular defect specimens obtained at 12 weeks were fixed in 4% paraformaldehyde for 24 h at 4 °C, and then trimmed using a band saw to remove the excess parts to evaluate the newly formed bone. The specimens were scanned using microfocus computed tomography (MILabs, Netherlands) at a voltage of 50 kV. The Amira software (USA) was used to analyze the image data and visualize the 3D images. The BV/TV, Tb.Th., and Tb.Sp. were quantified using ImageJ (NIH, USA), according to the literature.^57^ The samples that were scanned were decalcified in 50 mmol·L^−1^ EDTA, embedded in paraffin, and sectioned. The sections were stained using HE and Masson’s trichrome stain. The sections were stained with the following antibodies: anti-CD31 (Abcam, UK), anti-RUNX2 (Abcam, UK), anti-OSX (Abcam, UK), and DAPI (Vector, USA). Images were captured using an inverted microscope. New bone and vessels were analyzed using the ImageJ software (NIH, USA).

For RNA-seq test, fresh samples were taken from the defect area, carefully removed from the surrounding soft tissue, and were snap-frozen in liquid nitrogen. Total RNA was extracted using TRIzol (Gibco, USA), and RNA-Seq was performed by BioMarker (China).

This protocol was approved by the Ethics Committee of the Animal Care and Use Committee for Teaching and Research at Zhejiang University (ZJU20190154).

### Statistical analysis

All data are presented as the mean ± standard deviation (SD). Significant differences between the groups were analyzed using analysis of variance (ANOVA) and the Student’s *t* test.

## Supplementary information

supplemental material

The internal structure of HVEB
