# Supplementary material for: A hierarchical vascularized engineered bone inspired by intramembranous ossification for mandibular regeneration
Source: Int J Oral Sci. 2022 Jun 22;14:31. doi: 10.1038/s41368-022-00179-z (PMC9217949; doi:10.1038/s41368-022-00179-z)
Supplement: Supplementary file 1 — supplemental material [file 41368_2022_179_MOESM1_ESM.pdf]

Supporting Information

A hierarchical vascularized engineered bone inspired by intramembranous ossification for mandibular regeneration

Xin Ye, Jianxiang He, Shaolong Wang, Qianglong Han, Dongqi You, Bin Feng, Feiya Zhao, Jun Yin, Mengfei Yu,* Huiming Wang, Huayong Yang*

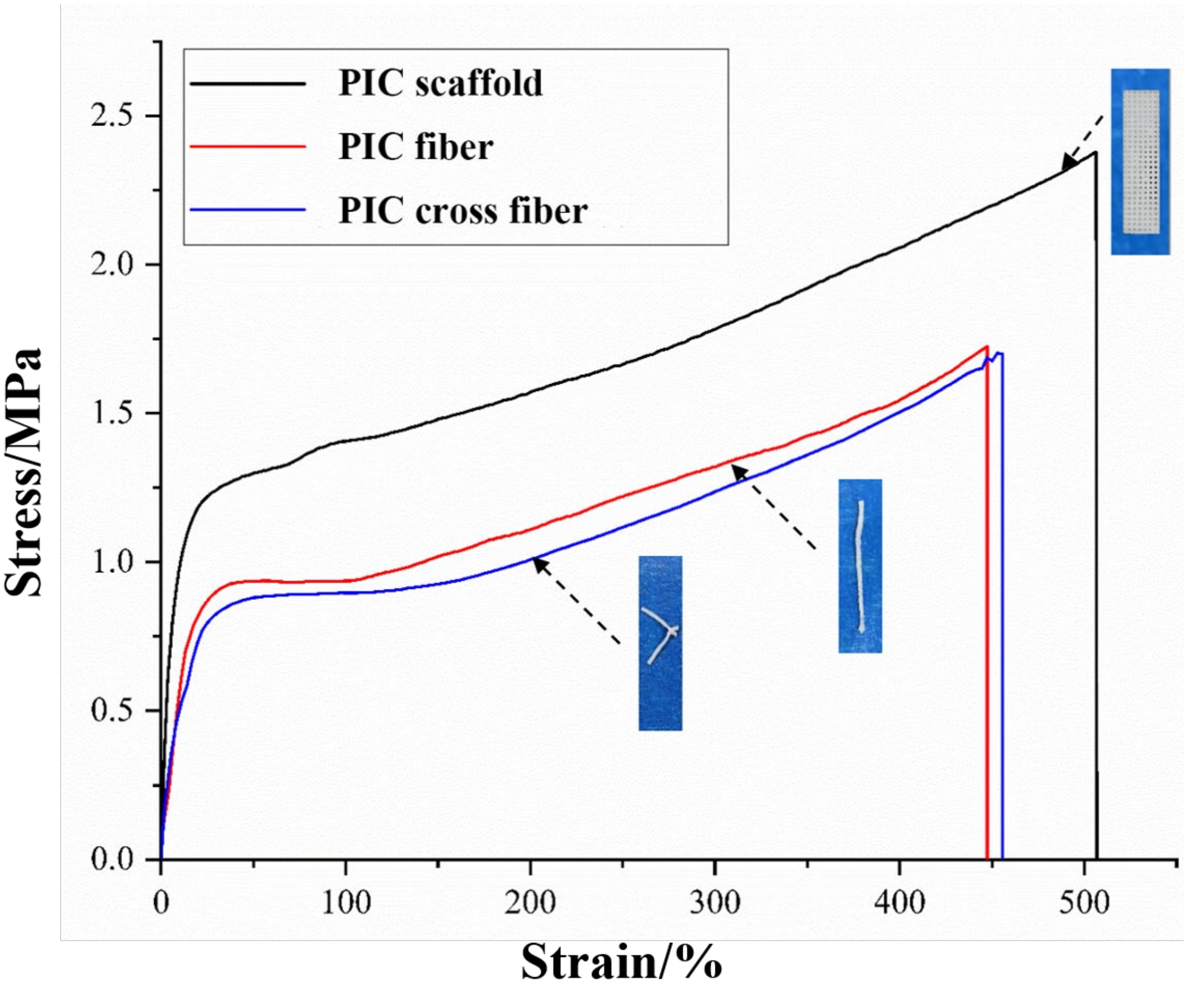

Supplementary Fig. 1. Tensile stress-strain curves of PIC fiber, cross fiber, and scaffold.

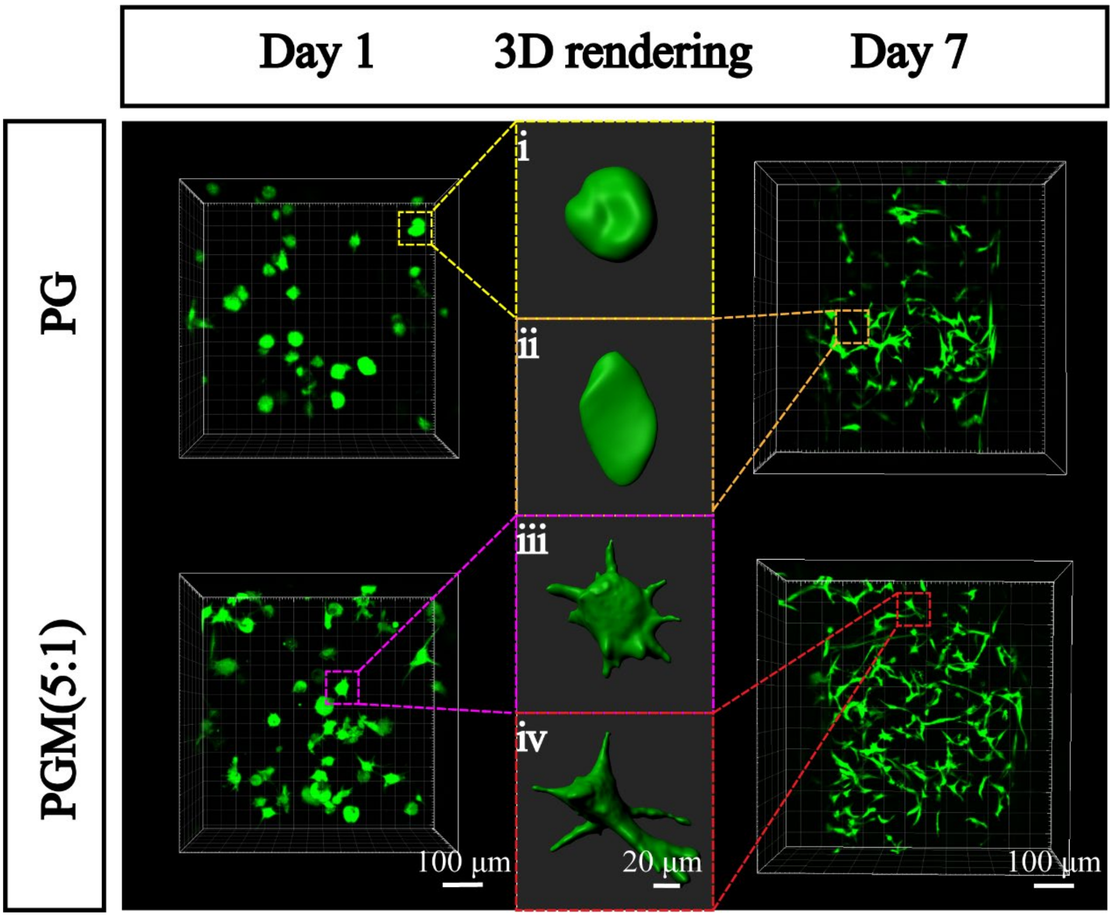

Supplementary Fig. 2. Representative LSCM images and 3D rendering images of MSCs cultured in PG and PGM (5:1) at day 1 and 7.

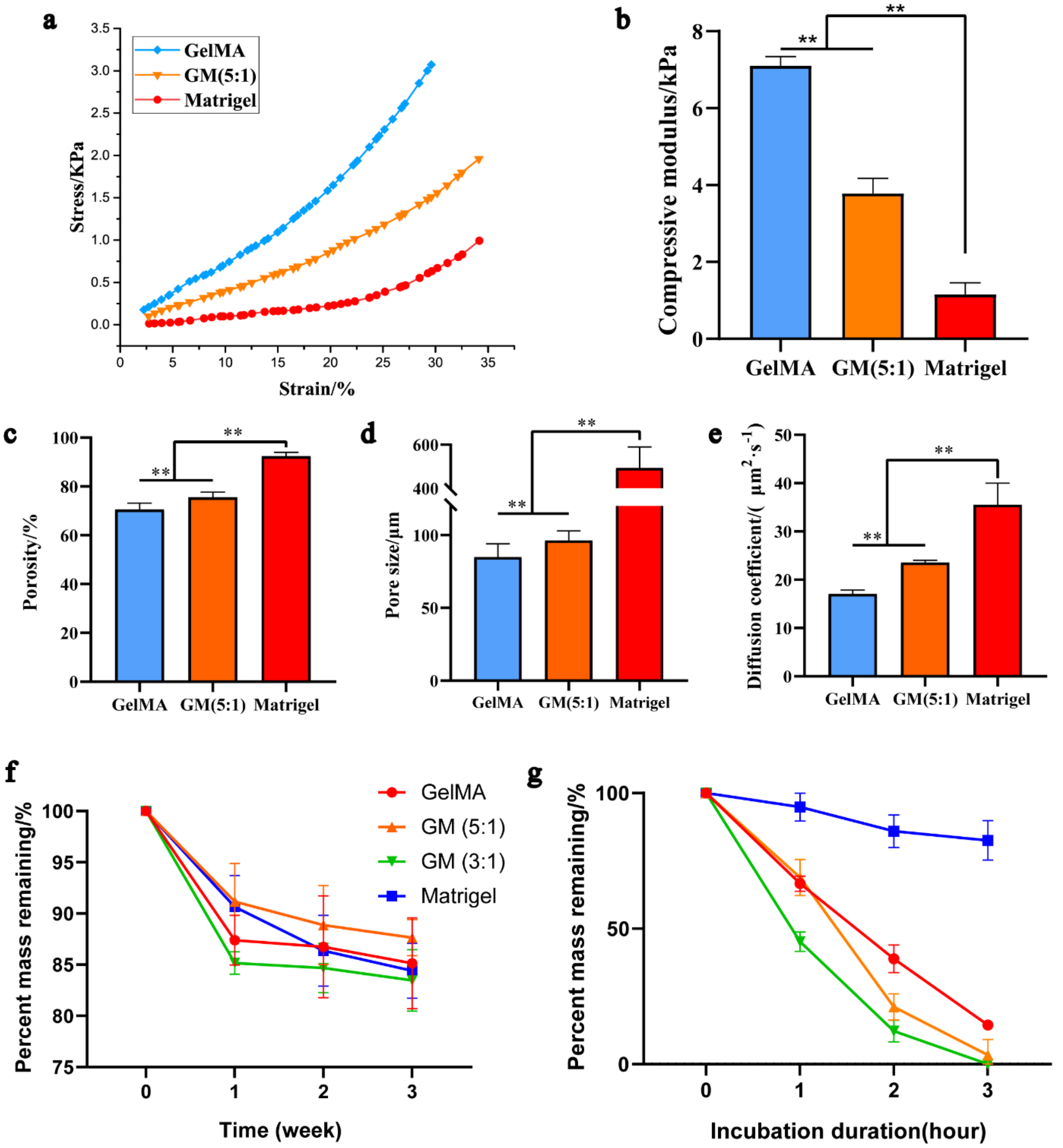

Supplementary Fig. 3. The mechanical properties and the microstructure of GM (5:1) are shown between that of GelMA and Matrigel. **a** Compressive stress-strain curves for GelMA, GM (5:1), and Matrigel. **b** Compressive modulus of GelMA, GM (5:1), and Matrigel (n = 3, $**P < 0.01$). **c** Porosity and **d** Pore size of GelMA, GM (5:1), and Matrigel (n = 3, $**P < 0.01$). **e** Diffusion coefficient of GelMA, GM (5:1), and Matrigel (n = 3, $**P < 0.01$). **f-g** The degeradation curves of GelMA, GM (5:1), GM (3:1), and Matrigel in PBS **f** and PBS with colleagense **g**.

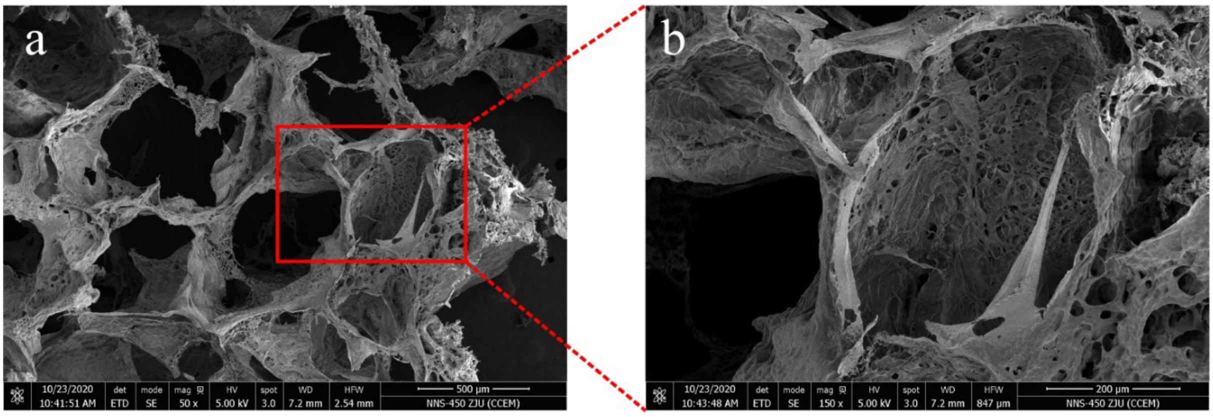

Supplementary Fig. 4. Low-magnification **a** and high-magnification **b** scanning electron microscopic images of Matrigel.

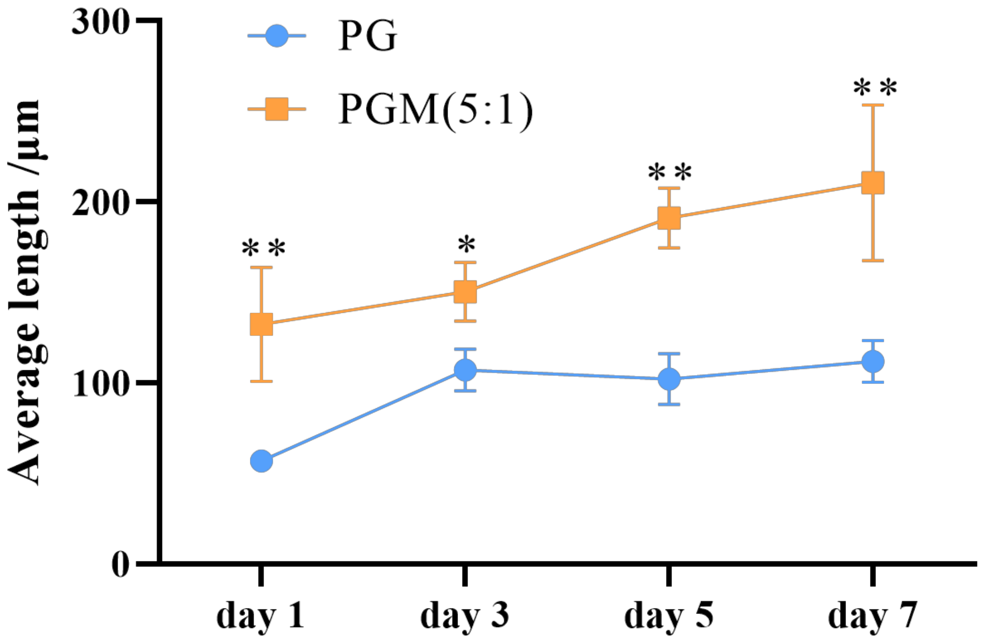

Supplementary Fig. 5. Average length of capillaries formed in PG and PGM (5:1) per unit of area (n = 4, *P< 0.05, **P < 0.01).

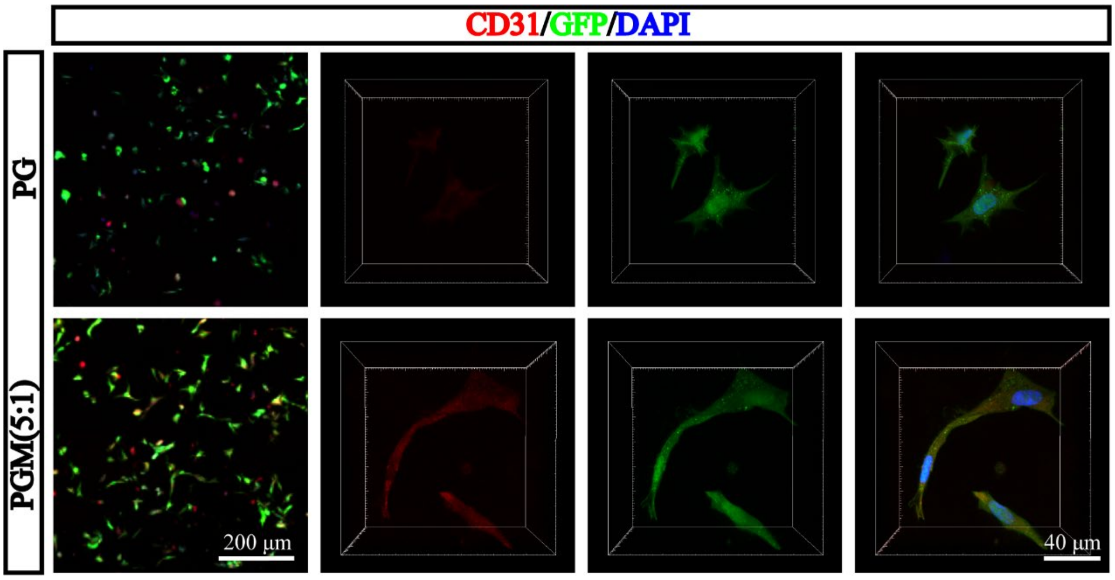

Supplementary Fig. 6. Representative immunofluorescence images of GFP-ECs (in green) stained with CD31 (in red) and nuclei (in blue) encapsulated in PG and PGM (5:1).

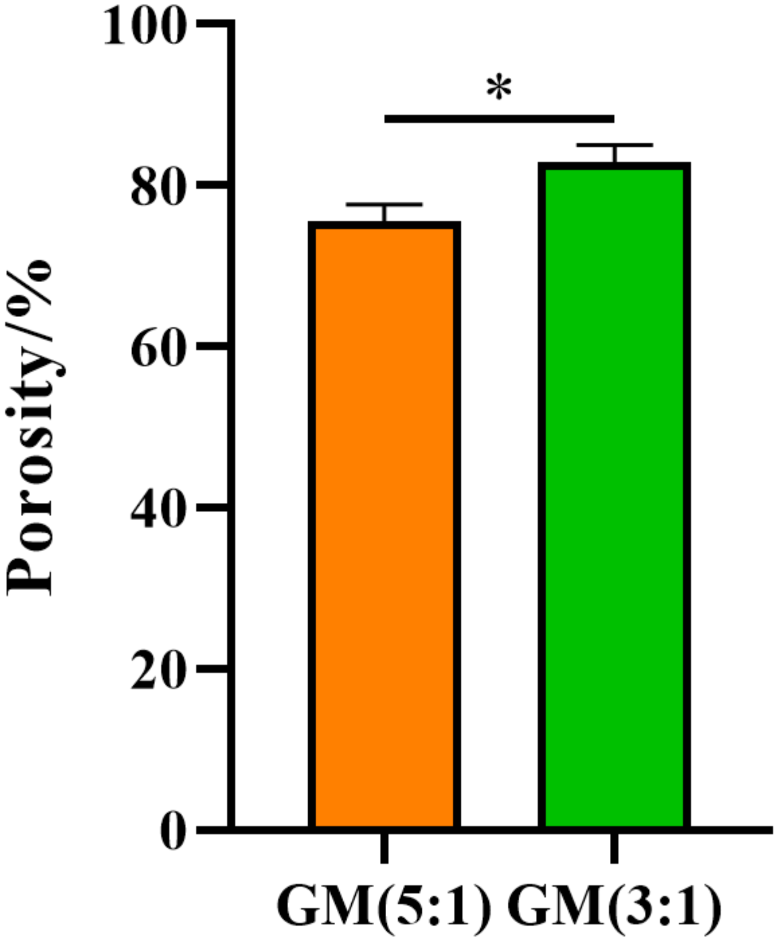

Supplementary Fig. 7. Porosity of GM (5:1) and GM (3:1). (n = 3, ***P*< 0.01)

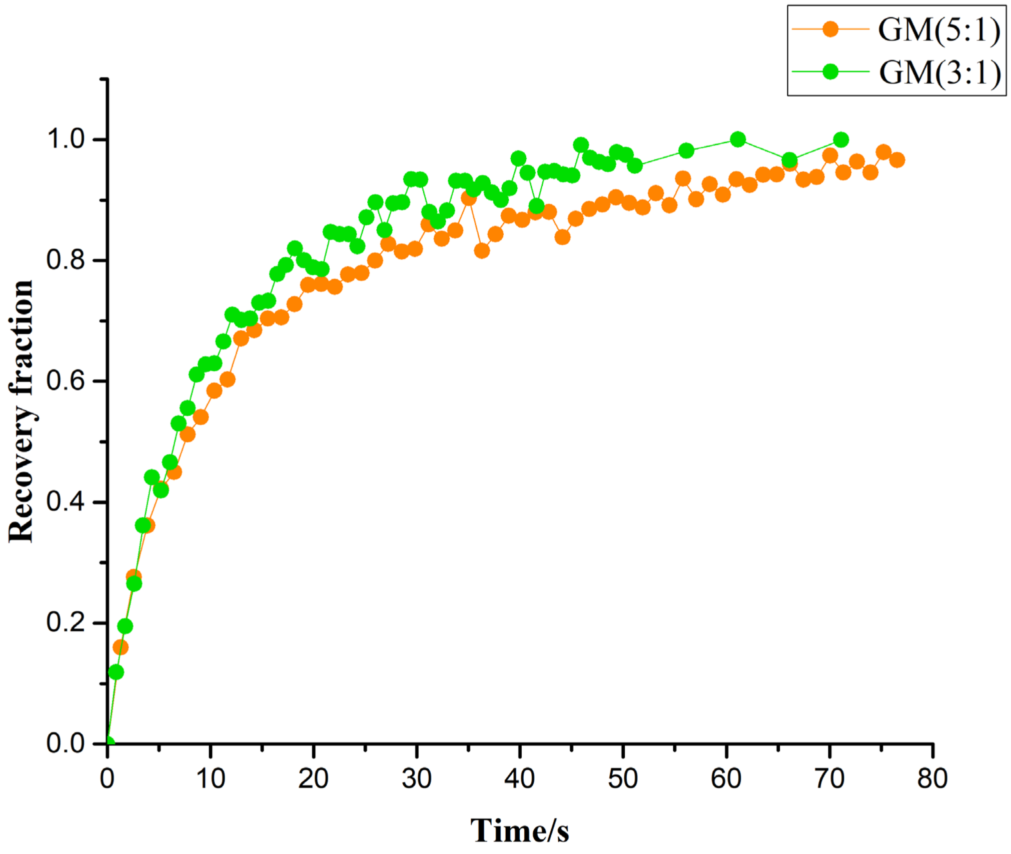

Supplementary Fig. 8. Recovery curves of GM (5:1) and GM (3:1).

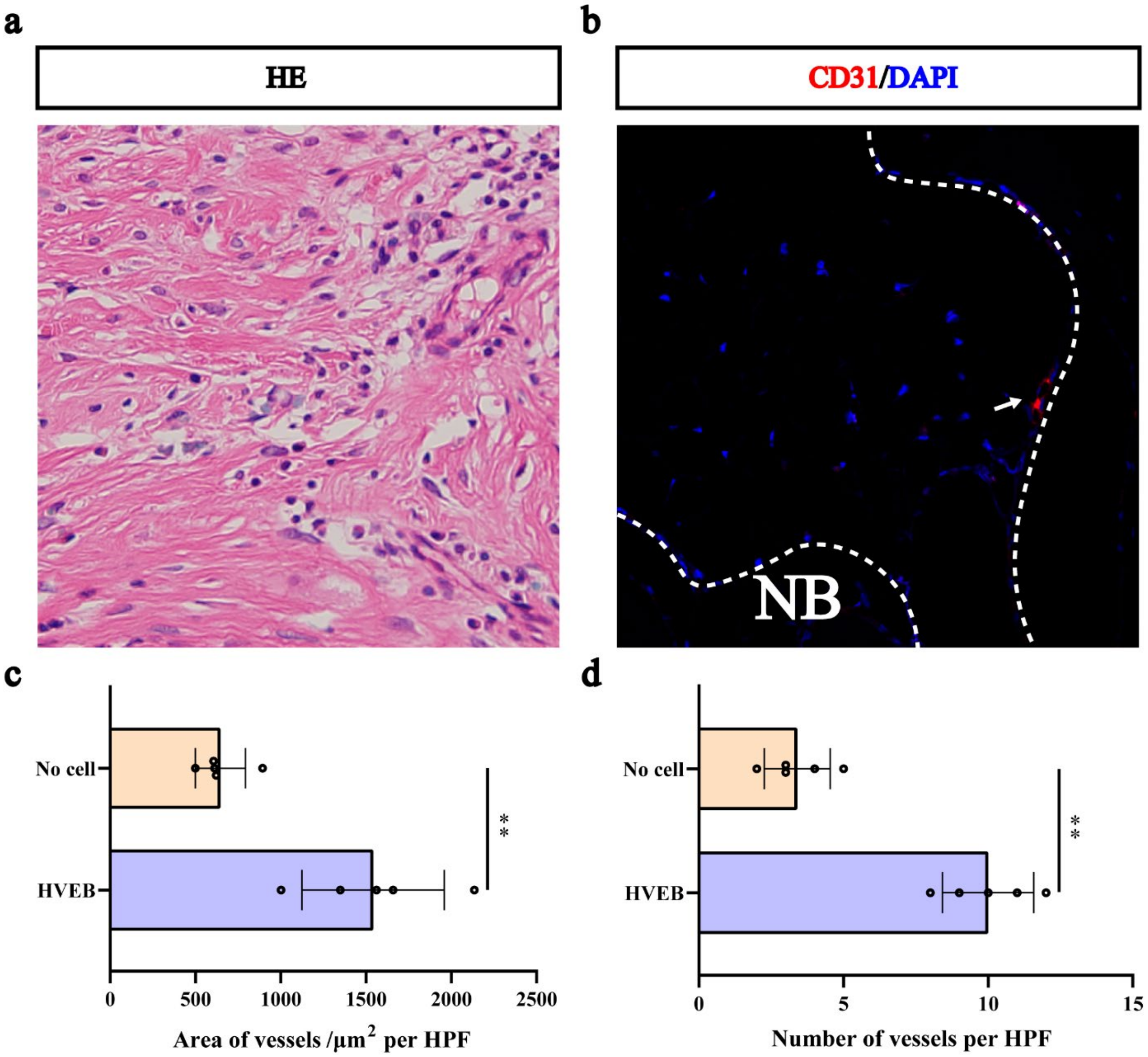

Supplementary Fig. 9. Vascular regeneration in the no cell group. **a** HE staining sections of neovascularization in the mandibular defect area. **b** Immunofluorescence images for CD31 of newly formed vessels. White arrows point to newly formed vessels. White dotted lines define the boundary of the newly formed bone. NB: new bone. **c-d** Quantification of area **c** and number **d** of newly formed vessels per HFP (x200). (n = 5, $^{**}P < 0.01$).

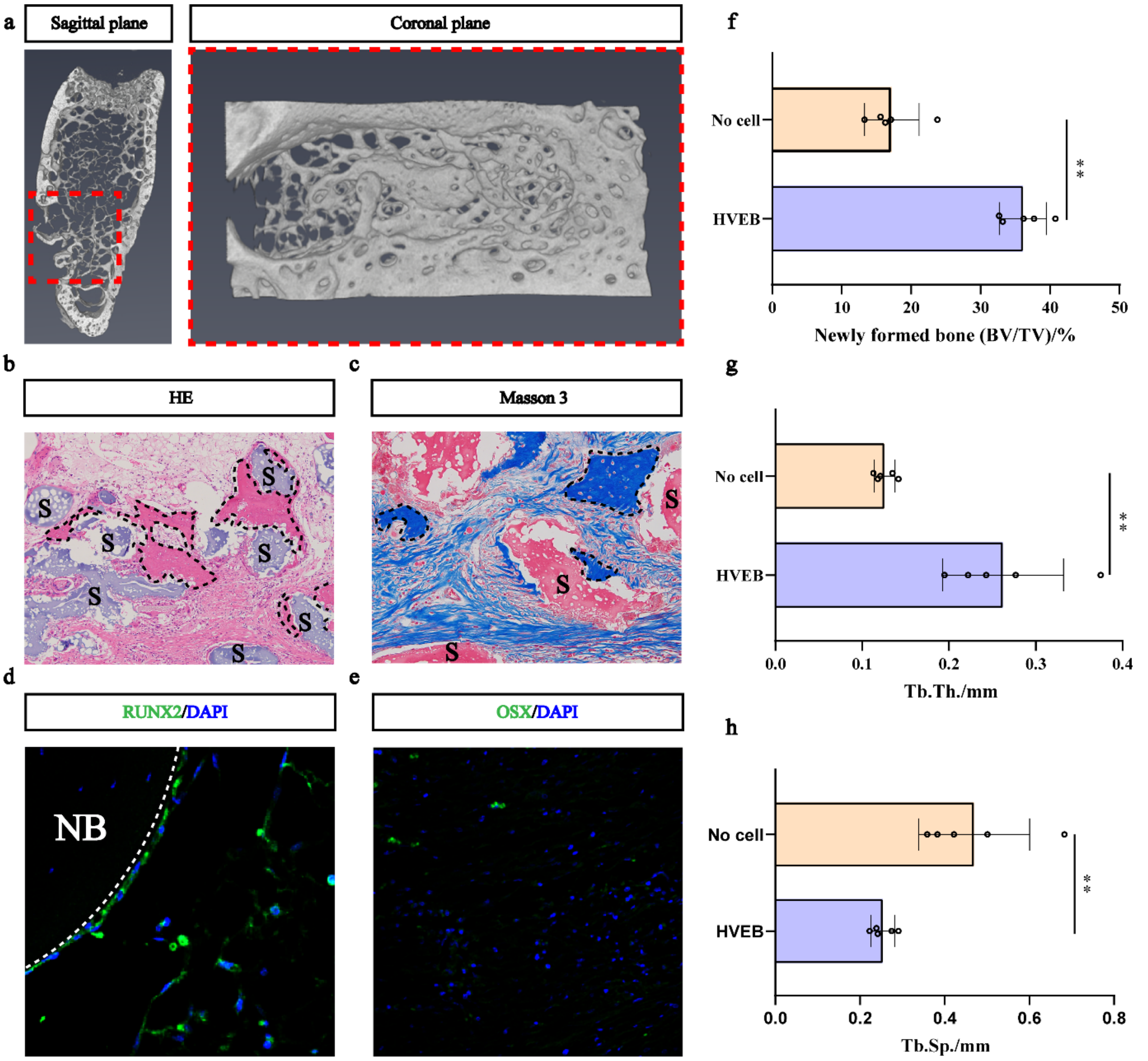

Supplementary Fig. 10. Bone regeneration in No cell group. **a** Representative 3D images of coronal sections in the no cell group. Red boxes define the defect margins. **b-c** HE **b** and Masson's trichrome **c** staining results of the bone regeneration in defect area. Black dotted lines define the boundary of the newly formed bone. S: PIC scaffold. **d-e** Representative immunofluorescence images of newly formed bone tissue stained with osteogenesis markers RUNX2 **d** and OSX **e**. White dotted lines define the boundary of the newly formed bone. **f-h** Quantified results of BV/TV **f**, Tb.Th. **g** and Tb.Sp. **h** from micro-CT. (n = 5, $^{**}P < 0.01$).

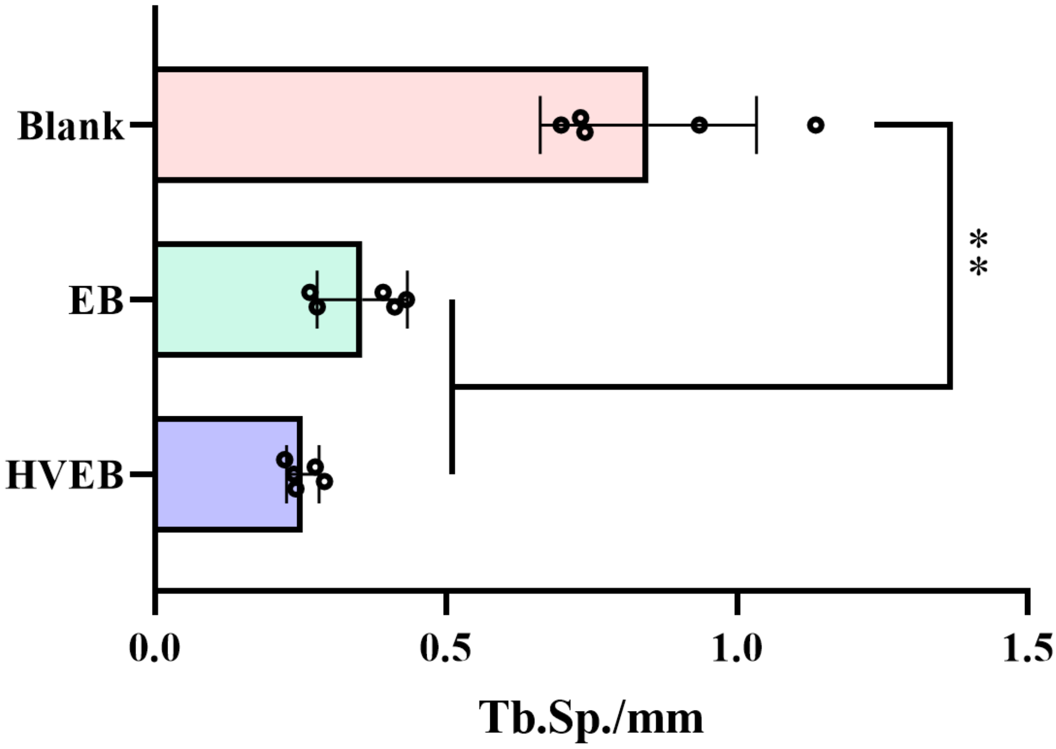

Supplementary Fig. 11. Quantified results of Tb.Sp. in Blank, EB and HVEB group from micro-CT (n = 5, ***P* < 0.01).

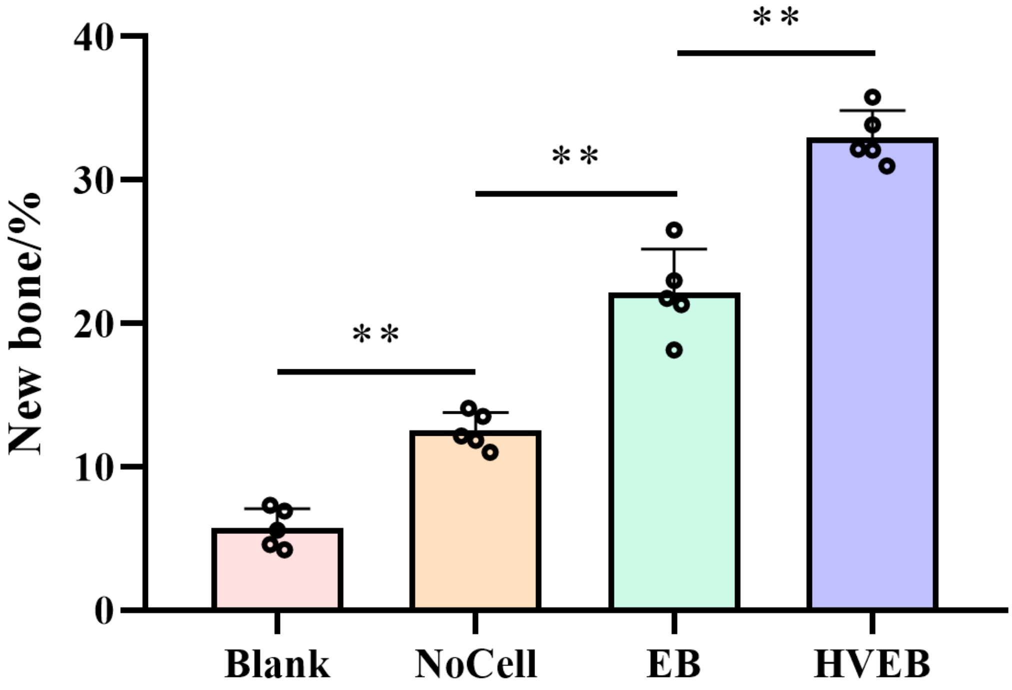

Supplementary Fig. 12. Quantified results of new bone formation from histological results (n = 5, ***P* < 0.01).

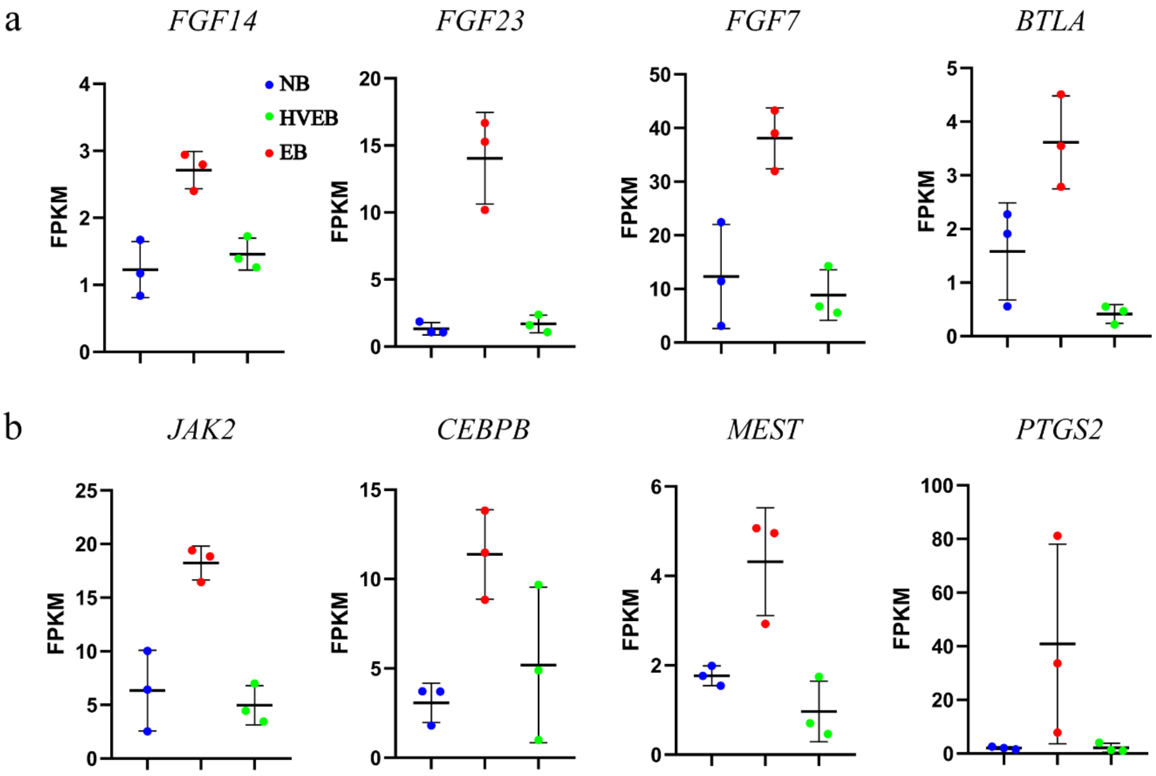

Supplementary Fig. 13. a Fibrogenesis gene signatures of *FGF 14, FGF23, FGF7*, and *BTLA* based on the relative expression levels in NB, EB, and HVEB groups. **b** Adipogenesis gene signatures of *JAK2, CEBPB, MEST,* and *PTGS2* based on the relative expression levels in NB, EB, and HVEB groups. (n=3)
